# The potential roles of JAK/STAT signaling in the progression of osteoarthritis

**DOI:** 10.3389/fendo.2022.1069057

**Published:** 2022-11-24

**Authors:** Qingluo Zhou, Qun Ren, Linhui Jiao, Jishang Huang, Jun Yi, Jincai Chen, Jinliang Lai, Guanglin Ji, Tiansheng Zheng

**Affiliations:** ^1^ Department of Orthopedics, First Affiliated Hospital of Gannan Medical University, Ganzhou, China; ^2^ College of Pharmacy, Gannan Medical University, Ganzhou, China

**Keywords:** osteoarthritis, JAK/STAT pathway, inflammation, cartilage remodeling, SOCS

## Abstract

Osteoarthritis (OA) is an age-related chronic progressive degenerative disease that induces persistent pain and disabilities. The development of OA is a complex process, and the risk factors are various, including aging, genetics, trauma and altered biomechanics. Inflammation and immunity play an important role in the pathogenesis of OA. JAK/STAT pathway is one of the most prominent intracellular signaling pathways, regulating cell proliferation, differentiation, and apoptosis. Inflammatory factors can act as the initiators of JAK/STAT pathway, which is implicated in the pathophysiological activity of chondrocyte. In this article, we provide a review on the importance of JAK/STAT pathway in the pathological development of OA. Potentially, JAK/STAT pathway becomes a therapeutic target for managing OA.

## Introduction

Osteoarthritis (OA), also known as degenerative arthritis or age-related arthritis, is a chronic progressive joint disease. The main pathological features include subchondral bone changes, inflammatory reactions, bone redundancy, and osteochondral angiogenesis (1–3). The cartilage tissue that makes up the joint consists of the only cell type and highly differentiated chondrocytes which maintain the homeostasis of the articular cartilage by regulating the integrity and biological stability of the cartilage (4). Importantly, chondrocytes are responsible for the synthesis of extracellular matrix (ECM) that mainly includes collagen and proteoglycans, maintaining the stability and integrity of the joint. The excessive death of chondrocytes and the consequent degradation of ECM are the central features of cartilage degeneration in the development of OA (5). It has also been suggested that the progression of OA is associated with the changes in biological actions of chondrocytes, including the proliferation, senescence, and apoptosis (6, 7).

As a multifactorial disease, OA is pathologically developed with a very complex process, and the risk factors include, but not limited to, aging, obesity, endocrine factors, genetics, trauma, and altered biomechanics (8–10). Of which, the biological and biomechanical factors have attracted the most interest in the academic fields (10, 11). For example, the excessive mechanical stress can induce biological alterations and apoptosis in chondrocytes (12–15). Increased levels of inflammatory cytokines are associated with inflammatory and immune responses in the joint cavity, causing alterations in the synovial membrane, muscles and cartilage (16–19). Current research suggests that cartilage damage is caused by a disrupted balance between the catabolic and anabolic capacities of chondrocytes (20, 21). Inflammatory responses are considered to be pathologically involved in this process, particularly in the early stage of OA (18). It has been shown that inflammatory synovitis is associated with damage in the cartilage (22). The stimulation of pro-inflammatory cytokines disrupts cartilage homeostasis and promotes the catabolism or degradation of cartilage (23).

The main therapeutic strategies for OA are to reduce pain and improve joint functions, enhancing the patient’s quality of life (24). Currently, physiotherapy, weight loss, non-steroidal anti-inflammatory drugs (NSAIDs), and hyaluronic acid (HA) injections are available for symptomatic relief in the early stages, and total joint replacement is often used for end-stage OA (25, 26). However, no cure strategies are available. This embarrassing situation might be due to the inexplicitly of the underlying molecular mechanisms in mediating the pathogenesis and progression of OA. The screening of specific therapeutic targets and the drug exploration for treating OA have become the challenge issues to be resolved.

## JAK/STAT signaling pathway

**Figure 1:**
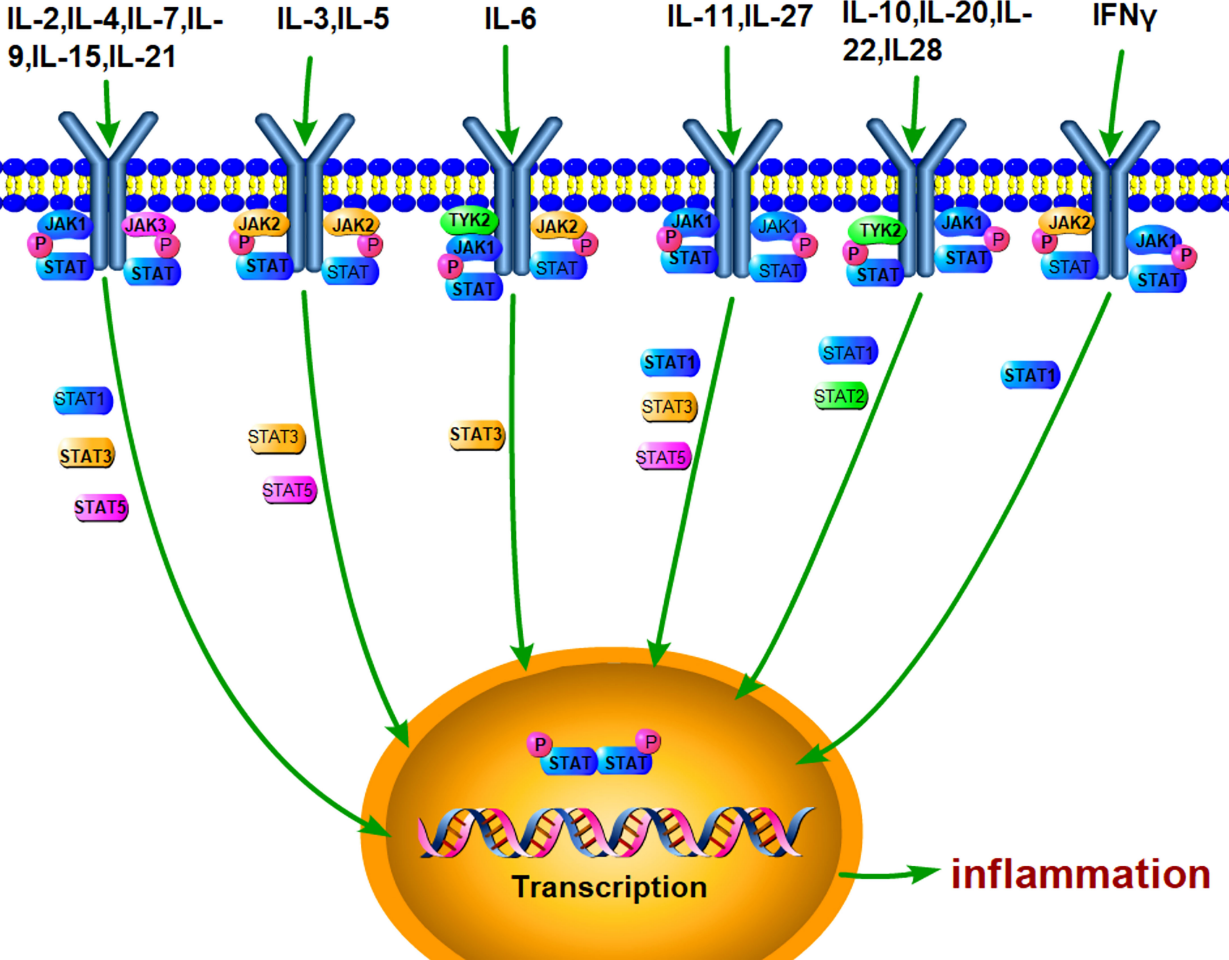
JAK/STAT pathway can be stimulated by various cytokines. A lot of extracellular cytokines, such as IL-6, IL-4, and IL-10, can interact with the specific receptors, which phosphorylate JAKs and recruit STAT. Activated STAT forms dimer and enter the nucleus for mediating the transcriptional expression of target genes, such as inflammatory cytokines.

Janus kinase (JAK)/signal transducer and activator of transcription (STAT) is an evolutionarily conserved signaling pathway, which can be stimulated by a variety of cytokines, interferons, growth factors, colony-stimulating factors, hormones, and other related molecules (**Figure 1**). Tyrosine kinase-associated receptors are the receptors located in cell membrane to specifically interact with cytokines or growth factors and then induce the activation of JAK by phosphorylating the tyrosine residues. Notably, this signaling pathway may accomplish signal transduction from extracellular factors to the nucleus (27, 28). It has been demonstrated that JAK/STAT signaling pathway is involved in many important physiological activities, such as cell proliferation, differentiation, immune regulation, and apoptosis (29–31).

JAK is a class of non-transmembrane tyrosine kinases in the JAK family consisting of JAK1, JAK2, JAK3, and Tyk2. Of which, JAK3 is mainly expressed in hematopoietic cells, while the other three are widely expressed *in vivo* (28, 32). The JAK molecule contains approximately 1000 amino acid residues and has seven internal homology domains (JH1-JH7) that form four distinct structural domains: a N-terminal FERM structural domain, a SH2 structural domain, a pseudokinase structural domain, and a protein tyrosine kinase structural domain. JH1 and JH2 are at the C-terminal, and JH3-7 is at the C-terminal. The kinase domain in each JAK is at JH1, and the pseudokinase structural domain is at JH2. The SH2 structural domain and FERM structural domain are at JH3-5 and J5-7, respectively (27).

STAT is a downstream target of JAKs and has seven members: STAT1, STAT2, STAT3, STAT4, STAT5a, STAT5b, and STAT6. The STAT protein mainly consists of a conserved N-terminal sequence, a DNA-binding region, a SH3 structural domain, a SH2 structural domain, and a C-terminal transcriptional activation region (TAD). The N-terminal structure is conserved and plays an important role in the phosphorylation and dimerization of STAT, while the C-terminal transcriptional activation region with conserved phosphorylated serine and tyrosine may recruit transcriptional activators to enhance the transcriptional activity by binding to the DNA binding region. The SH2 domain is also highly conserved in sequence and is a target of most STAT inhibitors. In addition, the SH2 domain possesses the sequence “GTFLLRFSS”, which is identical to the core sequence in the SH2 domain of the tyrosine kinase Src and contributes to protein-protein interactions (33, 34).

As one of the most important signaling pathways, JAK-STAT facilitates cytokine-mediated cell activation in a very simple and effective manner by eliciting a response to complete transmembrane receptor-to-nucleus signaling. Specifically, the various cytokines bind to their specific surface receptors and form dimers, which subsequently phosphorylate JAK kinase and then recruit the STAT protein. Phosphorylation and activation of STAT facilitate to detach from the receptor complex. Finally, STAT-STAT forms dimer, which is translocated into the nucleus for transcriptional regulation by binding to the specific DNA fragments. However, activated STAT dimers are dephosphorylated and inactivated after they conduct their functions in the nucleus, before being transported to the cytoplasm (27, 35).

Interestingly, the regulation of JAK-STAT signaling pathway in transcriptional outcome exhibits a great plasticity, due to the involvement of a broad set of intrinsic conditions. These include selectivity arising from the differential sensitivity of genes to STAT, interactions with other transcription factors, genomic competition, and the heterologous STAT signaling. Different STATs have their own regulatory pathways and physiological roles. For example, STAT1 is involved in antiviral and antibacterial responses, growth inhibition, apoptosis stimulation, and tumor growth inhibition (36–39). STAT2 has been reported the antiviral, immunomodulatory, antiapoptotic, and antiproliferative effects (40). In addition, STAT2 can also affect the functions of STAT3 by interacting with IL-6, thereby influencing cell proliferation and apoptosis (41). STAT3 can be activated by a number of cytokines. The most representative of which is IL-6, which induces a reduction in nuclear export signals and leads to nuclear aggregation of STAT3 (34). Meanwhile, STAT3 binds to the promoter of IL-6 and increase its expression, creating a positive feedback loop in the IL-6/JAK/STAT3 pathway (42).

STAT3 can also be activated by other pathways, such as hormones (growth hormone, prolactin, and leptin), growth factors (EGF, PDGF, FGF, and IGF), receptor-related kinases and non-receptor tyrosine kinases (Src and ABL), and Toll-like receptors. It has been reported that STAT3 can inhibit apoptosis and promote cell survival, and STAT3 deletion may lead to embryonic death (43–45). Consistently, transient STAT3 activation restores tissue integrity and promotes wound healing. However, sustained STAT3 activation is associated with mitogenesis, anti-apoptosis, metastasis and carcinogenicity (34). STAT3 regulates the transcriptional processes of downstream target genes through the regulation of growth factors. Activated STAT3 promotes tumor proliferation by increasing the expression of cyclin D1 and c-Myc and stimulates cell survival by enhancing the expression of Bcl-2, surviving, and Bcl-XL (34). STAT5 also plays an important role in maintaining intracellular organelles and regulating cell proliferation, cell differentiation, and survival in progenitor B and T cells (46). STAT5 up regulates the activity of PI3K/PTEN and HIF-1α signaling pathways, inhibits DNA damage, and protects against cell apoptosis, as demonstrated by down regulation of miRNA15/16 and up regulation of Bcl-2, MCL-1, and Bcl-XL expression (34).

## The implication of JAK/STAT pathway in OA inflammatory and immune responses

**Figure 2:**
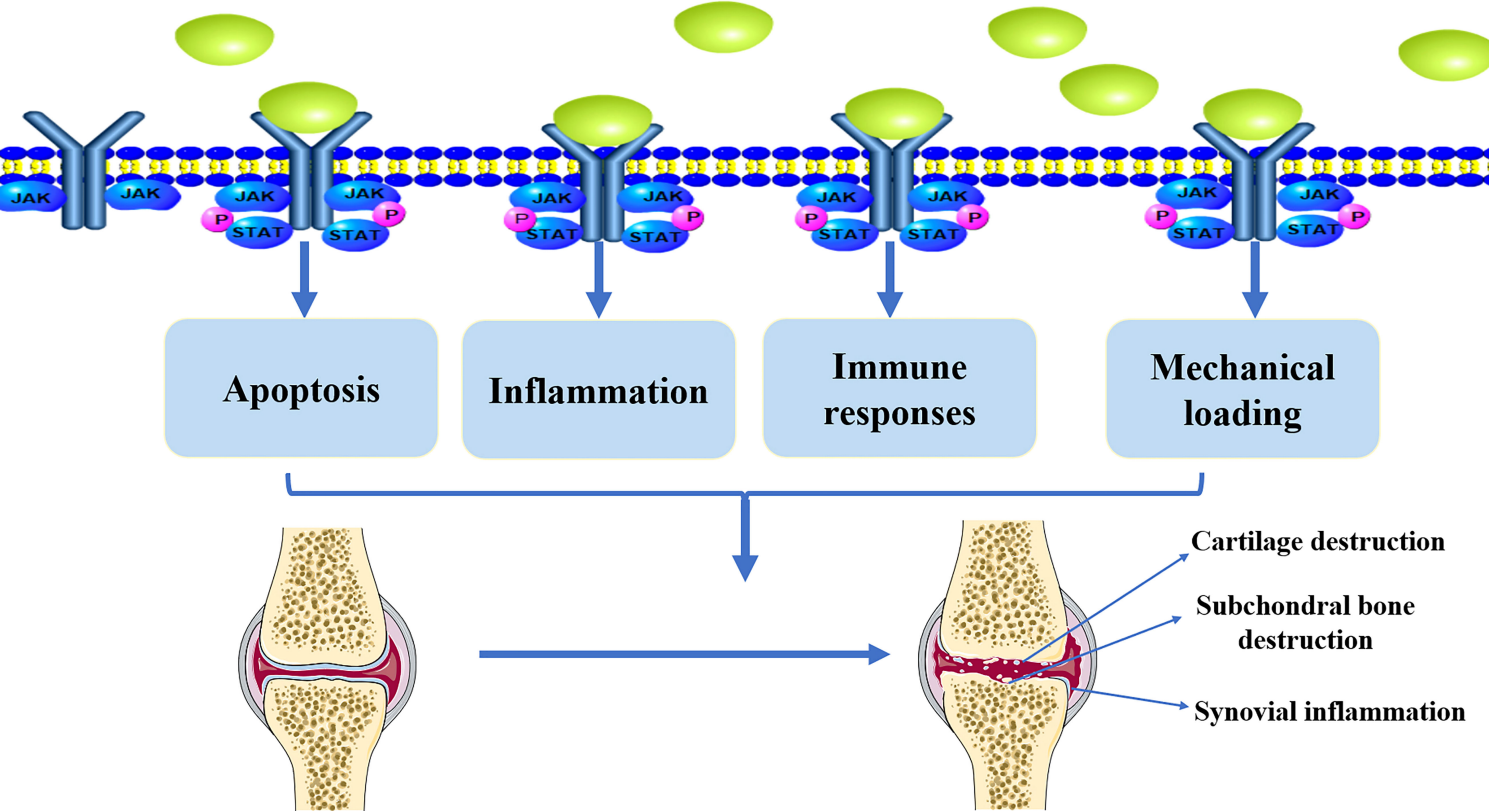
The implication of JAK/STAT pathway in the pathological development of OA. Active JAK/STAT pathway may induce the pathological development of OA by regulating inflammation, immune responses, mechanical loading, and apoptosis, leading to cartilage destruction, subchondral bone destruction, and synovial inflammation.

Inflammation plays an important role in OA pathogenesis. Pro-inflammatory factors such as tumor necrosis factor (TNF)-α, IL-1β, and IL-6 exhibit complex regulatory functions in OA development (47–49). For example, IL-1β induces the production of pro-inflammatory cytokines, such as IL-6, which promote catabolism and inhibit articular cartilage anabolism (50, 51). TNF-α can mediate the activation of matrix metalloproteinases (MMPs), which present in the ECM and promote cartilage destruction (52, 53). IL-6 activates MMPs and ADAMTSs, which are currently considered to be the major mediators of catabolism, altering the metabolic balance of chondrocytes and promoting cartilage degradation (54–57). Apoptosis is a tightly regulated process of programmed cell death. Apoptotic signaling pathways have been implicated in the development of OA. Pro-inflammatory factors, such as IL-1β, have been shown to induce apoptosis in chondrocytes, and higher levels of IL-6 in the serum and synovial fluid are correlated with OA progression (58–60). Reduced blood levels of IL-6 and IL-17 cytokines can ameliorate inflammatory events associated with OA (61). Pro-inflammatory cytokines, such as IL-6, can be the stimulators on JAK/STAT pathway. Thus, JAK/STAT pathway plays an essential role in the regulation of apoptosis, and it is closely associated with inflammation in the progression of OA (**Figure 2**).

IL-6 has been shown to induce JAK/STAT signaling pathway in multiple systems. The progression of OA is likely to be inextricably linked to IL-6/JAK-STAT pathway (62–64). In addition to IL-6, similar regulatory effects can be achieved by other inflammatory factors, such as TNFα, IL-4, IL10, IL13, IL17, and IL23. IL-4 has also been shown to exert anti-inflammatory effects in chondrocytes (65), and the JAK/STAT pathway is thought to play an important role in the regulation of IL-4 signaling (66, 67). IL-4 induces CITED2 expression in human chondrocytes *via* the JAK/STAT pathway, while CITED2 plays an important chondroprotective role by inhibiting MMP13 expression (68–71). Moderate mechanical loading has a protective effect on cartilage and synovial membranes. It has been found that the expression of phosphorylated JAK3 and STAT6 is increased by treatment with the combination of IL-4 and mechanical loading, suggesting that at least some of the combined effects of inflammation and mechanical stress on cartilage and synovium are mediated through the JAK/STAT pathway (66). Interaction of IL-10 with its two receptors IL-10R1 and IL-10R2 can activate JAK/STAT/SOCS signaling pathway (72). IL-23 can interact with FLS in joint inflammation and bone destruction with the assistance of STAT3 (73).

Macrophages are the most abundant immune cells in synovial joints and are the main innate immune effector cells that trigger the initial inflammatory response during the pathology of OA (74). In macrophages, full-length lipocalin induces the M2 phenotype *via* IL-4/STAT6/HO-1, reduces the sensitivity of macrophages to TLR4 ligand stimulation, and increases M2 markers (75). STAT1 and STAT3 have been shown to influence inflammatory responses in macrophages (76). Meanwhile, IL-13 and IL-4 were found to activate the M2 phenotype by activating the JAK2/STAT3 signaling pathway, with IL-4 phosphorylating STAT3 and STAT6 and up-regulating the DNA binding activity of STAT3, and IL-13 initiating the Tyk2 cascade of STAT1 and STAT6 and increasing the DNA binding activity of STAT1 (77). IL-6 can also activate the M1 phenotype of macrophages *via* the JAK2/STAT3 signaling pathway (78, 79). In previous studies it has been found that there is some overlap between OA and RA patients with elevated inflammatory factors such as TNF-α, IL-1β, IL-6 (80–84).

Study has been shown that elevated M1/M2 ratio in macrophages is highly correlated with OA severity (85). Classically, activated M1 macrophages are associated with a high production of pro-inflammatory factors as well as chemokines, including IL-1, IL-6, IL-12, IL-23, and TNF-α. Excessive inflammatory responses can cause cartilage damage and destruction, induce joint pain, and aggravate OA progression (86). On the contrary, M2 macrophages may leads to down regulation of IL-12, IL-23, IL-1, IL-6, and TNF-α, triggering anti-inflammatory and immunosuppressive responses and playing a role in pathogen clearance, wound healing, tissue remodeling, and immune regulation. Potentially, enhancement of M2 macrophages can counter the pathological changes of OA (87). STAT6 activation and nuclear translocation contribute to the polarization of M0 into M2 macrophages (88). One study has been shown that quercetin can ameliorate ECM degradation in OA models by down regulating STAT6 signaling in a time-dependent manner, promoting M2 macrophage production, and inhibiting inflammatory responses (89). Squid Type II Collagen (SCII) has also been found to promote the polarization process towards M2 macrophages by negatively affecting the phosphorylation of STAT6 and the nuclear translocation of p-STAT6 in OA cartilage (87). Thus, increasing the number of M2 macrophages, reducing the proportion of M1 macrophages, and interfering with STAT6 activation may result in improvement of cartilage homeostasis and OA development. However, more efforts are still needed on this issue.

## JAK/STAT pathway participates cartilage remodeling

The pathological development of OA involves the remodeling of subchondral bone (90–92). There is also evidence that aberrant activity of transforming growth factor-β (TGF-β) in subchondral bone can induce abnormal recruitment of mesenchymal stem cells (MSC), leading to development of subchondral osteosclerosis (92). The subchondral bone and articular cartilage work together to coordinate joint stress and maintain joint stability, and their interaction is inextricably linked to the occurrence and development of OA (3, 93–95). It is possible to counteract OA through the protection of subchondral bone. The levels of MMP isoforms in synovial fluid and subchondral osteocytes were significantly elevated in patients with OA (96). CDC42 may cause deterioration of subchondral bone and induce cartilage degradation through JAK/STAT activation (97). In addition, muscle loss can be related to anabolic resistance in OA, where muscle wastage disrupts joint homeostasis (98, 99). STAT3, a muscle transcription factor, has been shown to be associated with joint dysfunction in OA patients (100). This suggests that JAK/STAT may also affect joint homeostasis in terms of subchondral bone and muscle loss (**Figure 2**).

A study has been shown that JAK2/STAT3 pathway is involved in the reduction of collagen II in chondrocytes (101). In addition, JAK/STAT3 activation in chondrocytes can be induced by IL-1β, which promotes MMPs expression (102, 103). JAK3 expression is also up regulated in mechanical loading-treated and IL-4-treated chondrocytes (66). A study suggests that CXCL8 and CXCL11 may be involved in apoptosis and inhibit the proliferation of primary chondrocytes through up regulation of phosphorylated STAT3 expression (104). A series of studies have been demonstrated the possibility of inflammatory factors regulating the OA process through the JAK/STAT pathway, providing a possible direction for the development of targeted drugs. It has been shown that TGF-β1 can protect chondrocytes from IL-6 catabolism by limiting STAT3 phosphorylation and blocking SOCS3 induction (105). In addition, the formation of a complex between Smad3 and STAT3 might be involved in the pharmacological activity of activated TGF-β1 in protecting OA chondrocytes (106, 107). TGF-β also induces hypertrophic differentiation of chondrocytes during OA progression with a possible mechanism of potentiating STAT3 expression (108).

JAK/STAT pathway has been involved in the promoting effects of IL-6 in up regulating the expression of MMP1, MMP3, and MMP13 in human chondrocytes (109, 110). Consistently, JAK1/STAT3 activation and interaction with ERK pathway in bovine articular chondrocytes is associated with loss of matrix (55). In DMM-induced mice OA models, STAT3 and ERK1/2 expression are activated in cartilage, and inhibition of IL-6/STAT3 signaling suppresses ECM remodeling and osteophyte formation (57). In addition, JAK2/STAT3 can stimulate the nuclear translocation of NF-κBp65, up regulate NF-κB pathway, trigger the expression of RANKL, and increasing the expression of MMP-3 and MMP-9 in arthritis cartilage (111). In intervertebral disc chondrocytes, IL-21 may increase the expression of STAT-1, STAT-3, and STAT-5b and promote the expression of MMP-13 and ADAMTS-7, leading to the degeneration of intervertebral disc. Treatment with STAT3 inhibitor AG490, the expression of MMP-13 and ADAMTS-7 is down regulated, and the degenerative activity of intervertebral disc is ameliorated (112). These suggest the roles of JAK/STAT pathway in mediating cartilage remodeling, which is associated with the expression of ECM degrading enzymes.

## The roles of SOCS/JAK/STAT in the development of OA

SOCS family contains seven members, including SOCS1, SOCS2, SOCS3, SOCS4, SOCS5, SOCS6, and SOCS7. SOCS protein consists of a N-terminal binding domain, a SH2 domain, and a proteasome-interacting and ubiquitin-associated SOCS box domain. SOCS1-3 provides a feedback regulation of cytokine signaling *via* the JAK/STAT pathway (113). SOCS1 and SOCS3 may inhibit the activity of JAK1, JAK2, and TYK2, but not JAK3. SOCS 4-7 regulates growth factor receptor signaling (27). Activated STATs may promote SOCS gene transcription, and the interaction between SOCS and phosphorylated JAK or JAK receptors may block JAK/STAT pathway (114). For example, the binding of SOCS1 to JAK3 can inhibit gp130-mediated pathway and negatively regulate STAT (115, 116). Similarly, SOCS3 can inhibit the phosphorylation of both JAK and STAT3, blocking the stimulation of JAK-STAT3 pathway (34, 35). This suggests the possibility of SOCS as a therapeutic target for OA.

The negative effects of SOCS against JAK/STAT in the pathological development of OA have been discussed recently (117). The involvement of SOCS3 has been demonstrated in the attenuation of carboxymethyl chitosan-induced inflammatory responses in chondrocytes by inhibiting IL-10 (118). TGF-β1 protects chondrocytes against IL-6-induced catabolism by mediating SOCS3/STAT3 (105). The accumulation of AGEs in the joints has been associated with weakness and stiffness. AGE-mediated activation of MMP-13 and reduction of type II collagen and proteoglycan can be attenuated by blocking JAK/STAT signaling (101, 119). Silencing of TBK1 down regulates ADAMTS-4, MMP3 and MMP13 expression while up regulates SOX9, collagen II, and aggrecan expression, thereby attenuating ECM degradation and cartilage degradation. These effects can be counteracted by pcSTAT3 (120).

It has been shown that leptin can promote the expression of IL-6, MMP-1, MMP-3, and MMP-13 and decrease the expression of SOCS3. Knock down of SOCS3 can induce inflammatory responses and enhance leptin-mediated MMP-3 expression in OA chondrocytes (121). In addition, oncostatin M (OSM) induces inflammatory responses, which is highly associated with IL-6 and orchestrated by gp130 in chondrocytes. SOCS3 can restrain gp130-mediated transcriptional alterations in inflammatory activity in chondrocytes (122). In SOCS3 knock-out mice, reduced proliferative chondrocytes and decreased proliferative zone width in the growth plate are observed. This pathological change might be associated with altered expression of FGFR3-mediated MAPK signaling pathway in chondrocytes (123). Furthermore, FGFR3 activation stimulates the expression of STAT1 and MAPK, and prolonged STAT1 activation induces a dysregulated response in chondrocytes proliferation. SOCS3 may mediate FGFR3 pathway by decreasing downstream MAPK expression, preventing inhibition of chondrocyte proliferation (123, 124).

## The involvement of microRNA in the regulation of JAK/STAT pathway in OA

Gene modification to affect protein expression is also a novel therapeutic strategy. CircRNA can influence the transcription of miRNAs and thus the expression of downstream mRNAs, with implications for the disease process (125). MiR profiling also revealed differential expression between OA and other diseases. This suggests that they may serve as prospective diagnostic markers and therapeutic targets for OA (126). A series of related studies have provided a degree of evidence for the therapeutic effects. For example, circRNA_0092516 deletion promotes chondrocyte proliferation through the miR-337-3p/PTEN axis and impedes apoptosis, thereby improving OA (127). JAK/STAT is associated with the expression miR-224 in osteogenic differentiation. Conversely, miR-224-5p can also promote cartilage degradation and aggravate OA by activating JAK2/STAT pathway (128, 129). miR-149-5p has been shown to be an early biomarker of OA, and JAK inhibitors exhibit therapeutic effects against OA through inhibiting the expression of miR-149-5p (130). LOC101928134 inhibits synovial proliferation and cartilage destruction in OA rats by inactivating JAK/STAT pathway (131).

MiR-149/JAK1/IL-6/TNF-α axis plays a key role in maintaining joint tissue homeostasis. Specifically, miR-224-5p activates JAK2/STAT pathway by targeting CCL2, thereby promoting cartilage degradation and exacerbating symptoms in OA patients (129). In contrast, tofacitinib (a JAK inhibitor) affects miR-149 expression in C28/I2 cells and inhibited JAK/IL-6/TNF-α pathway, reducing arthritis scores and bone degradation in mice (130). ZNF667-AS1 has been reported to increase cell proliferation and suppress IL-6, IL-17, and TNFα expression in LPS-treated chondrocytes by inactivating JAK/STAT signaling pathway. miR-523-3p can specifically interact with ZNF667-AS1 and abolish its inhibitory effects on JAK/STAT signaling pathway (132). miR-223 can enhance LPS-induced inflammatory responses by activating the activity of JAK2/STAT1 pathway in intervertebral disc chondrocytes, increasing the expression of MMP-3, and promoting ECM degradation (133). Bioinformatics analysis has been reported that hypoxic treatment can increase the repairment of OA cartilage by promoting the proliferation and migration and suppressing the apoptosis of chondrocytes through miR-18/JAK/STAT pathway (134).

## Future perspectives

IL-6 and its soluble receptor sIL-6R can promote cartilage degradation through the induction of ADAMTS-4, ADAMTS-5/11, MMP-1, MMP-3, and MMP-13, which might be regulated by STATs (109, 135). Conversely, disruption of IL-6/sIL-6R binding may decrease the expression of MMPs, reduce the degradation of ECM, and thus alleviate the progression of OA. Inactivated IL-6 gene completely can protect mice from collagen-induced arthritis or delay the onset or reduce the severity of the pathological process (136, 137). In addition, rhIL-6 activates STAT3 in C-28/I2 chondrocytes and increases MMP production, which may be associated with the degradation of ECM and the destruction of articular cartilage (138). Treatment with anti-mouse IL-6R antibody also inhibits the development of arthritis in DBA/1J mice and protects the knee joints from damage (139).

It has been demonstrated that JAK inhibitors can maintain joint tissue homeostasis by modulating the effects of IL-6 (130). JAK/STAT pathway has been involved in the pathological changes of both OA and RA. There is a rationale and clinical significance for the development of therapeutic agents for OA *via* JAK. Current anti-rheumatic drugs have been found to modulate the activation of JAK/STAT pathway (140). Blockade of either JAK2 or JAK3 reduces the expression and enzymatic activity of MMP-13, ADAMTS-4, and ADAMTS-5 and prevents the reduction of collagen II (101). It has been suggested that JAK inhibitors can also rescue chondrogenic differentiation and promote cartilage regeneration by stimulating the actions of mesenchymal stem cells (MSC) (141). Similarly, the JAK inhibitor AG490 can inhibit JAK2/STAT3 pathway, suppressing IL-1β-induced expression of interferon regulatory factor 1 (IRF-1) and ameliorating the course of OA (142). STAT3 expression is associated with joint dysfunction and disability. It has been found that both JAK1 and JAK2 inhibitors block TNF-α-induced STAT3 phosphorylation and the binding of STAT3 to DNA (143).

Chinese medicine has been used for treating OA for a long time. Many Chinese herbal medicines have been demonstrated the efficacy against OA, but most of them are explained with unclear mechanisms (144–146). By targeting JAK/STAT pathway, effective herbal ingredients that can improve OA symptoms or even reverse the process have been explored. Artesunate has been shown to mediate osteoclast formation and OA progression by inhibiting JAK/STAT signaling and pro-inflammatory cytokine expression (147). In addition, convallatoxin can promote apoptosis by regulating JAK2/STAT3 and mTOR/STAT3 pathways in colorectal cancer cells (148). Curcumin has also been found to promote apoptosis in retinoblastoma through the regulation of JAK/STAT pathway (149). B6, a naturally occurring compound, can interact with the upstream kinase JAK2 *via* its FERM-SH2 structural domain to induce apoptosis (150). Osthole may also promote apoptosis and inhibit the growth of gallbladder cancer cells by inactivating JAK/STAT3 pathway (151). Acteoside (ACT) can inhibit IL-1β-induced expression of inflammatory factors, such as IL-6, IL-12, and TNFα, and apoptosis in chondrocytes by decreasing the expression of JAK/STAT pathway (152). Although most of them are unknown to be effective against OA development and what the exact mechanisms are, they at least provide us with a large number of possible potential therapeutic agents for OA management.

## Conclusion

**Figure 3:**
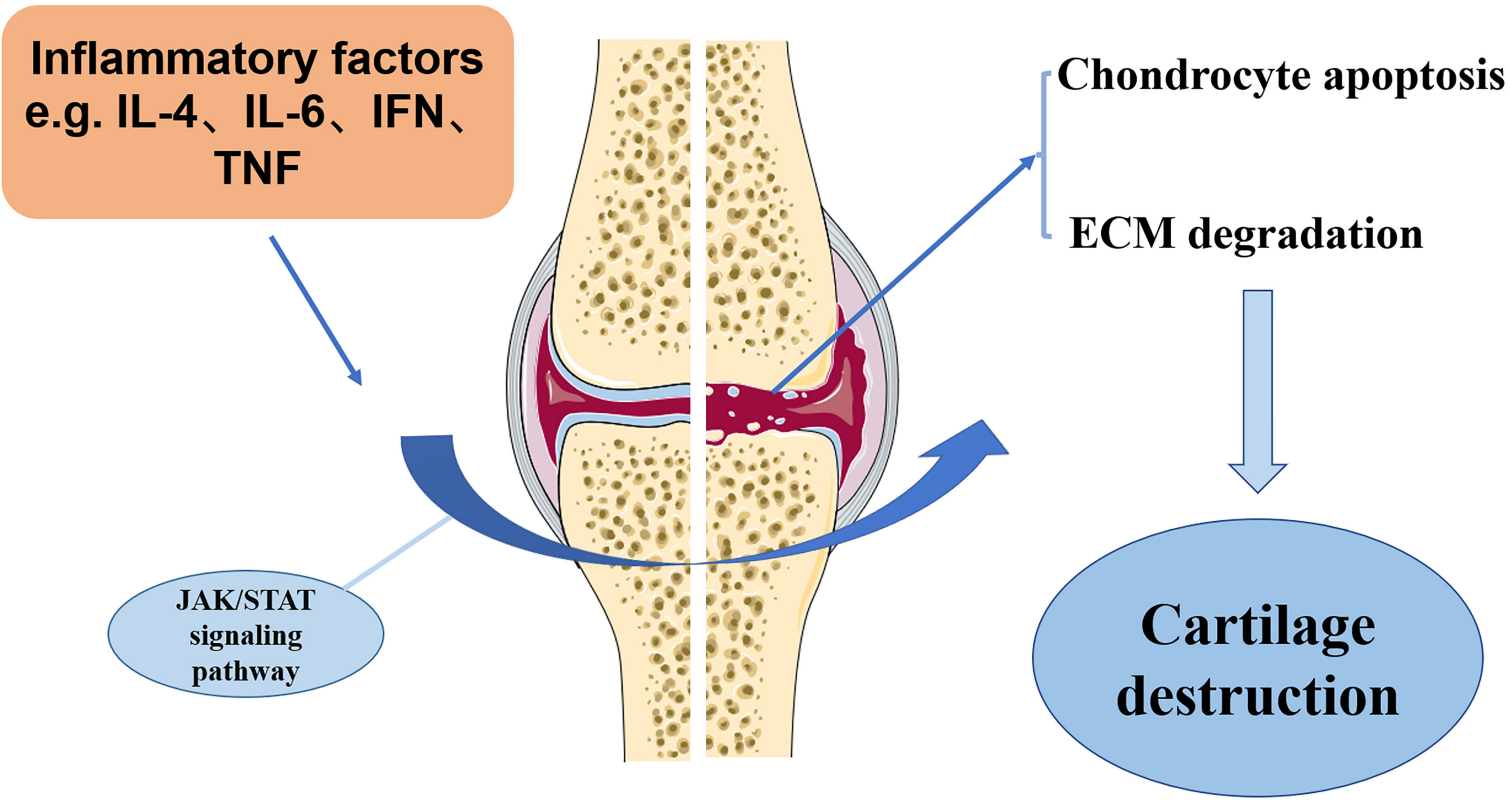
The potential mechanism of JAK/STAT pathway in OA development. Inflammation factors, such as IL-6, IL-4, and TNFα, in the OA cartilage can stimulate JAK/STAT pathway, which induces the destruction of OA cartilage, as shown by increased chondrocyte apoptosis and ECM degradation.

OA, a multifactorial disease with complex etiology, affects joint’s functions throughout the body by inducing pain and even physical disability. The current treatments for OA are mostly symptomatic and lack an effective cure. It is therefore essential to investigate the specific molecular mechanisms underlying the pathogenesis of OA and to develop new therapeutic targets. There is considerable evidence that JAK-STAT pathway plays an important role in the development of OA. Pro-inflammatory factors-associated JAK/STAT signaling pathway has been demonstrated to be critical for orchestrating the inflammatory responses in the pathological development of chronic inflammatory diseases, such as OA. However, current investigation has been focusing on unraveling the possible molecular mechanism of JAK/STAT pathway in regulating OA chondrocyte survival, apoptosis, repair activity and ECM degradation. Perturbations in JAK-STAT pathway may result in various pathological changes, which are crucial to many of OA clinical aspects. JAK-STAT pathway as the critical mediators of MMP gene expression. It is essential to detect the extent to which JAK inhibitors alter the expression of MMP in chondrocytes. Overall, JAK-STAT pathway promotes ADAMTS- and MMP-mediated ECM degradation and reduces type II collagen expression in chondrocytes (**Figure 3**). It is essential to seek effective strategy against JAK/STAT pathway, which has become a therapeutic target for management of OA.

## Author contributions

TZ and GJ provided the idea of this paper. JH, QZ, JY, JC, JL, QR, LJ, and CT conducted the experiments and revised and finalized the paper. All authors approved the final paper.

## Funding

This study was financially supported by Science and Technology Plan of Jiangxi Health Commission (220210619, 202210897 and 202210900), Guiding Science and Technology Plan Project of Ganzhou City (GZ2021ZSF021), and Gannan Medical College Science and Technology Innovation Construction Team (TS202002).

## Acknowledgments

We thanks Dr. Chunfang Tang for her help in evaluating our manuscript.

## Conflict of interest

The authors declare that the research was conducted in the absence of any commercial or financial relationships that could be construed as a potential conflict of interest.

## Publisher’s note

All claims expressed in this article are solely those of the authors and do not necessarily represent those of their affiliated organizations, or those of the publisher, the editors and the reviewers. Any product that may be evaluated in this article, or claim that may be made by its manufacturer, is not guaranteed or endorsed by the publisher.
